# Dentin Matrix Metalloproteinases: A Futuristic Approach Toward Dentin Repair and Regeneration

**DOI:** 10.7759/cureus.27946

**Published:** 2022-08-12

**Authors:** Paridhi Agrawal, Pradnya Nikhade, Manoj Chandak, Anuja Ikhar, Rushikesh Bhonde

**Affiliations:** 1 Department of Conservative Dentistry and Endodontics, Sharad Pawar Dental College and Hospital, Datta Meghe Institute of Medical Sciences University, Wardha, IND

**Keywords:** growth factors, signalling molecules, dentin repair, mmps, dentin regeneration, dentin matrix metalloproteinases

## Abstract

Matrix metalloproteinases (MMPs) have been linked to modulating healing during the production of tertiary dentin, as well as the liberation of physiologically active molecules and the control of developmental processes. Although efforts to protect dentin have mostly centered on preventing these proteases from doing their jobs, their role is actually much more intricate and crucial for dentin healing than anticipated. The role of MMPs as bioactive dentin matrix components involved in dentin production, repair, and regeneration is examined in the current review. The mechanical characteristics of dentin, especially those of reparative and reactionary dentin, and the established functions of MMPs in dentin production are given particular attention. Because they are essential parts of the dentin matrix, MMPs should be regarded as leading applicants for dentin regeneration.

## Introduction and background

Regeneration of the tissue must closely resemble parent dentin due to the firmly associated structural and functional relationship in the physiologic dentin. In other words, maintaining the mechanical characteristics of the tissue provided by its biological structure is necessary for dentin regeneration. Dentin is an essential mineralized tissue that contains odontoblasts' biological functions within dentinal tubules and is in charge of reducing mastication pressures [1]. These pressures need to be transmitted from a rigid (96% mineral by weight enamel) to a much more elastic (70% mineral by weight dentin) substance. Collagenous (86% type 1, together with types 3, 5, and 6) and non-collagenous proteins make up the dentin matrix. Following pulpal injury from cavity preparations, carious lesions, erosion, and restorative dental materials, dentin is capable of limited healing. The circumpulpal dentin layer grows inward as a result of dentin healing by tertiary dentin deposition, enlarging the pulp chamber and root canals. The exact tubular structure of physiological dentin is lost in tertiary dentin. Reactionary dentin forms in the shape of tubular odontodentin or atubular and bone alike osteodentin after minor trauma, which does not affect the underlying Hoehl’s cells or odontoblast layer [2]. Despite being substantially mineralized, reactionary dentin is less elastic and rigid [3]. Progenitor cells are needed to fill up the deficiency after more severe injuries that cause cellular death by laying reparative dentin in the shape of a dentinal bridge. The quantity of tertiary dentin in a tooth may or may not have an impact on how well it functions.

Dentin hardness is linearly correlated with tissue's lack of elasticity and directly proportional to the density of minerals present in the tissue. The tissue has different mineral densities, with peritubular dentin being the dentin that is closest to the dentinal tubule border, hardest, and least elastic [4]. The much more elastic intertubular dentin is found between tubules. Hardness is highest in circumpulpal dentin, lowest in the DEJ, and again falls toward the pulp [5]. Dentinal tubules increase in number, density, and size as they grow nearer to the cell body of odontoblasts [6]. Variations in the ratio of intertubular to peritubular dentin, which affects the tissue's hardness and mechanical qualities, are correlated with changes in dentinal tubule density. The effects of matrix metalloproteinase (MMP) activity on tube density and the structural and mechanical characteristics of tertiary dentin have not been demonstrated in research to our knowledge. Future regenerative models ought to take into account using these elements as success indicators because of how crucial they are to dentin function.

## Review

Dentin regeneration and its implications

The vitality of the pulp is always necessary for dentin repair and regeneration. This idea has been applied in the management of deciduous teeth that have open apices using regenerative endodontics. The center of the tooth is made up of the dental pulp, which is a loose connective vascular tissue. In close proximity to dentin, it is made up of cellular elements such as immune cells, odontoblasts, neuro-vascular networks, fibroblasts, and extracellular elements such as glycoproteins and collagen [1]. Vital pulp therapies help to regenerate vascularized and innervated dental pulp that can facilitate odontoblast differentiation and dentin neogenesis for the full development of roots [7]. Such a sort of dentin regeneration falls short of addressing the clinical crown loss brought on by caries. The amount of dentin that might be replaced in studies is unclear, as is the question of whether this would balance the amount lost via disease. Enamel is an acellular tissue; hence regeneration of this tissue presents much greater difficulties than dentin regeneration [8]. At the very least, tissue regeneration requires the capacity for cellular and matrix replacement through proliferation [9]. Replacement of the damaged dentin matrix by freshly generated, proliferating odontoblasts and differentiated dental pulp stem cell (DPSC) is necessary for dentin repair. In this regard, scaffolds and biological cues hold the most potential, enabling cellular infiltration, matrix deposition, and mineralization later on. Collagen, silk, chitosan, alginates, hyaluronic acid, hydrogels, and fibrin have all proved successful in promoting dental pulp cell maturation [10]. Despite the fact that these scaffolds claim to encourage pulpal regeneration, no research has demonstrated meaningful amounts of mineralization to compensate for the dentin that has been lost to caries. Additionally, the length of time needed to repair the bulk of the tissue that caries destroyed would be excessive and clinically inappropriate. To avoid massive forces from interfering with the process of regeneration, the tooth might need to be removed from its occlusal position. Additionally, a full-coverage restoration would definitely be necessary for a better long-term outlook.

Dentin MMPs

**Figure 1:**
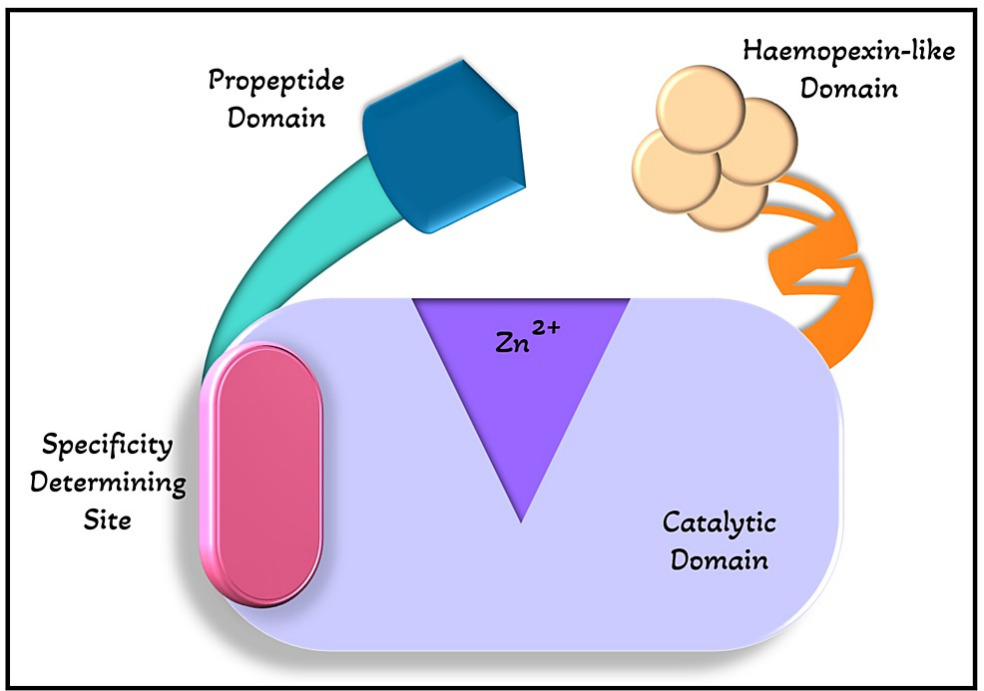
Structure of MMPs MMPs contain a zinc-binding catalytic domain, an specificity determining site, a propeptide domain, and a hemopexin-like domain.

MMPs are a group of 28 modular endopeptidases that play an important function in the remodeling of extracellular matrix and the control of extracellular signaling networks that, among other things, regulate inflammation, bone growth, and angiogenesis [11]. The cellular elements of both hard and soft tissues, such as epithelial cells, fibroblasts, osteoclasts, osteoblasts, as well as hypertrophic chondrocytes, chondroclasts, inflammatory cells, and odontoblasts, create these substances [12]. The majority of MMPs have a propeptide domain, which is in charge of maintaining the enzyme's latent conformation, a zinc-binding catalytic domain, which is in charge of their proteolytic function, and a hemopexin-like domain, which is in charge of protein-protein interactions as shown in Figure 1.

**Figure 2:**
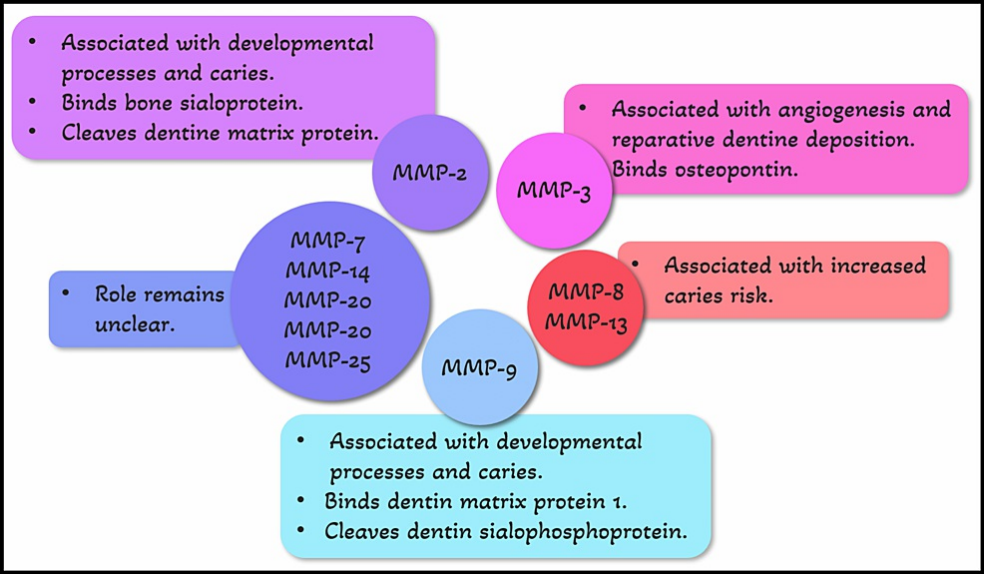
MMPs present in dentin and their functions

Various proteolytic and also nonproteolytic methods trigger these zymogens, which enables them to perform the many tasks that they are intended for [13]. Their substrates can be used to categorize them, and these substrates are mostly defined by specificity-determining sites on their catalytic domain [14]. MMP-2, -3, -7, -8, -9, -13, -14, -20, -23, and -25 are MMPs that are present in dentin as shown in Figure 2 [12,15-17].

MMP activation and inhibition dynamics are still poorly understood, despite being one of the significant aspects of tissue remodeling in reaction to illness. MMP’s proteolytic activity has previously been linked to tissue deterioration. A significant rise in the activity of MMP-14 has also been linked to a carious response, though its precise function is still unknown. MMP-8 has been recognized among the principal collagenases in human dentin linked to carious lesions [3]. These endogenous MMPs perpetuate the illness via enzymatic activity they have and could be produced from the extracellular matrix or triggered by the process of caries. Additionally, like in the case of MMP-13, their diminished capacity has been linked to lowered decay risk [16]. Inhibiting MMP activity has been the main focus of efforts to delay or prevent illness. It has been determined that MMPs' enzymatic destruction of collagen fibrils is what causes resin-based restorations to fail to owe to hybrid layer degradation. Exogenous MMP inhibitors have enhanced clinical results of the restorations, which are resin-bonded so far by maintaining the bond strength and hybrid layer. Examples include tetracycline antibiotics and chlorhexidine [18]. In human dentin, endogenous Tissue Inhibitors of Metallo Proteinases I to IV (TIMP-I, TIMP-II, TIMP-III, and TIMP) have been found. Even though this increase occurs at the same time as raising MMP expression, TIMP-II expression rises during the caries process [3,12]. Despite the fact that the MMP/TIMP ratio and the substrate-inhibitor specificity might help to describe and regulate the activity of MMPs in tissues, the importance of their co-expression has not yet been shown. It has also been suggested that TIMP signaling occurs independently of its MMP-inhibitory effect. Important elements of scaffolds utilized in regenerative endodontic operations include TIMP-I and TIMP-II expression [19]. Additionally, it is known that substances that promote pulp cell proliferation also increase the expression of TIMP [20]. In these circumstances, MMP counter-regulatory effect on TIMP signaling may explain MMP co-expression. In the end, matrix turnover must be balanced for regeneration. Knowledge of MMP-TIMP protein interactions and biological processes is necessary for maximizing MMP activity.

MMPs are essential for tooth growth. The earliest MMPs to express are MMP-2 and MMP-9, which may help with basement membrane breakdown and signal the beginning of ameloblast and odontoblast terminal differentiation [21]. MMP-2 (and MMP-20) loss of function causes larger levels and a better reach of non-collagenous proteins known to stimulate dentin mineralization, which is significant at early stages [22]. Still, as development progresses, root dentin abnormalities are caused by the dysfunction of other MMPs, such as MMP-14 [17]. MMPs are not absurd proteins with similar roles, according to the available evidence. Their unique modes of action have the potential to influence tissue development, and their imbalance has important consequences for tissue integrity. The propensity for cell maturation, as well as the necessary remodeling of freshly deposited dentin matrix, are required for dentin regeneration and tertiary dentin creation. For these processes, MMPs are essential. In order to achieve regeneration, it may not be possible to totally stop their proteolytic activity. In fact, even though they are present throughout the disease, some MMPs may operate as a protective mechanism by aiding in the healing process. The optimum proteolytic activity and calcium affinities of MMP-3 are pH-dependent (range pH 5.2-5.6). Thus, at the essential pH for demineralization of enamel and dentin, as those observed in carious settings, its proteolytic activity is at its peak [23]. However, angiogenesis and reparative dentin deposition have both been linked to MMP-3 [24].

Utilizing MMPs to promote dentin regeneration

MMPs have been linked to the control of developmental processes, the release of physiologically active molecules, and the modulation of repair during the production of tertiary dentin, among other things [25]. MMPs open pathways for progenitor cells to enter and also activate growth factors that control angiogenesis, the immune system, and cellular differentiation. When a disease is present, MMP activity may be produced by endogenous (immune cells), exogenous (bacterial products), and dentin matrix reservoirs. Bacterial byproducts from decayed lesions may also activate odontoblast MMP release by activating signaling cascades. High MMP-2 expression in odontoblasts next to reactive dentin has been linked to higher proteolytic activity in this region [26]. The maturation of collagen fibers and the beginning of mineral production in the freshly created dentin may be caused by this action [27]. MMPs aid in the removal of detritus and the development of tissue. The significance of these enzymes in the regenerative process is supported by animal models of tissue regeneration. One of the first steps in new limb regeneration following amputation is MMP overexpression. MMP activity is necessary for limb regeneration, and its suppression slows it down [28]. The most highly expressed MMPs in this particular model were MMP-9, MMP-3, and MMP-13 [29]. Similar to other injuries, pulp damage triggers an inflammatory response that includes the invasion of PMN’s cells and the production of proteases like MMP-9 [30]. These MMPs aid in the breakdown of exposed carious dentin, angiogenesis, and cell migration, which activates the processes that result in the deposition of tertiary dentin [26]. As a diagnostic and predictive indicator of pulpal inflammation, MMP-9 is being employed in endodontics to help direct clinical decisions [30].

Because MMP-3 has anti-inflammatory characteristics, it can reverse mild irreversible pulpitis in vivo. These characteristics include a reduction in the invasion of macrophages and antigen-presenting cells, as well as the suppression of Interleukin-6 (IL-6) production [31]. Additionally, independent of its proteolytic activity, MMP-3 can increase the synthesis of the connective-tissue growth factor (CTGF), which promotes the migration of dental pulp cells [32]. In vivo pulp damage models, MMP-3 is also localized to endothelial cells and promotes angiogenesis and reparative dentin deposition [24]. The lack of increased MMP-3 activity in pulps with irreversible damage supports the protein's potential for regeneration [33]. Future models of dentin regeneration should pay close attention to MMP-3 as a regeneration mediator. A palisade layer of odontoblasts that lines the pulp chamber's perimeter is in charge of producing dentin. These cells release bioactive chemicals during dentin deposition that direct the tissue's mineralization. Similar to this, these cells are prompted to secrete new dentin in response to dentin breakdown brought on by attrition, carious exposures, and chemical assaults. The chemical signals and cellular dynamics necessary for primary dentin production, however, are not present during regeneration and repair. As a result, the tissue is dependent on bioactive chemicals to promote the differentiation and proliferation of cells necessary for dentin regeneration and repair [34].

The bioactive chemicals that were once engaged in the natural deposition of mature dentin are stored in the tissue. As a result, the tissue has a defense system against environmental assaults. Dentin contains sequestered forms of Transforming Growth Factor-1 (TGF-1), Platelet-Derived Growth Factor- AB (PDGF-AB), Vascular Endothelial Growth Factor (VEGF), Placenta Growth Factor (PlGF), and Fibroblast Growth Factor-2 (FGF-2) [35]. These growth hormones are made soluble, which encourages angiogenesis, odontoblast differentiation, and tertiary dentin deposition. As a result, dentinal bridges that are denser, thicker, and more structurally similar to physiological dentin are produced [36]. Additionally, removing these components from plasma can maintain the viability of tooth-bud cells, which has led to the regeneration of teeth in porcine animal models [37]. A promising method for releasing growth factors and activating the genes that promote odontoblast differentiation is dentin conditioning with ethylenediaminetetraacetic acid (EDTA) [38]. Another way to release these elements from tooth tissues is through MMPs. These proteases may be exposed while still serving their purpose when phosphoric acid etch-and-rinse adhesive methods are used [39]. Numerous of these proteases have been linked to the promotion of pulp repair. Similar regeneration qualities have been shown in vivo using direct pulp capping agents made of dentin matrix components that have undergone MMP digestion [40]. These investigations recognized MMP-1, MMP-9, MMP-13, and MMP-20 as pulpal healing boosters. In tissue regeneration models, endogenous MMP activity has been used to transport growth factors from scaffolds. In recent work, Huang et al. [41] constructed a scaffold including growth factor-binding sites and an MMP-2 cleavage site. When activated by MMP-2 in vivo, such hydrogel scaffolds would be helpful for the release of growth factors.

It has been demonstrated that concentrated venous blood growth factors, including PDGF-BB, TGF-1, VEGF, and others, can stop dental pulp cells from releasing proinflammatory cytokines and stimulate the regeneration of dentine-pulp complex in vivo [42]. Melatonin-induced dental pulp TGF - secretion has also been used to immunomodulate the pulpal inflammatory response to damage [43]. By attracting dental pulp stem cells, promoting their proliferation and odontogenic differentiation, as well as promoting pulp angiogenesis, overexpression of PDGF-BB encourages regeneration [44]. Similar to Bone Morphogenetic Protein (BMPs), FGF-2 and bioactive pulp-capping agents like BMP-2 and BMP-4 promote cell differentiation and tertiary dentin deposition [45]. The tissue engineering trio includes scaffolds, signaling molecules, and cells. Successful scaffolds must endure in tissues for a sufficient amount of time to permit cellular colonization before being degraded by enzymes [46]. In order to maintain cell colonization and long-term proliferation, two factors necessary for neovascularization and angiogenesis, MMPs have been used to remove hydrogel scaffold systems at the appropriate moment [47]. The scaffolds must be kept in place by the native cells that are present in the regenerating tissues. Anti-inflammatory cytokines like IL-10 that are released cause TIMP expression and stop MMPs from degrading the scaffold [48]. On the other hand, it is also known that inflammatory cells in the regenerating tissues secrete MMPs that modify these scaffolds and freshly deposited extracellular matrices [49]. As was already mentioned, MMPs play a crucial role in the development of tertiary dentin. These enzymes enhance the bioavailability of signaling molecules, the cellular processes that result in dentin healing, and the maturation of the dentin collagen matrix. In the mineralization process, activation of signaling molecules is crucial. MMPs play a role in the maturation and function of a class of non-collagenous proteins known as small integrin-binding ligand, N-linked glycoprotein (SIBLINGs), in addition to the collagenous components of the dentin matrix. This family of proteins contains the proteins Osteopontin (OPN), dentin-matrix protein-1(DMP-1), dentin sialo phosphoprotein (DSPP), matrix extracellular phosphoglycoprotein (MEPE), and bone sialoprotein (BSP-2). Both latent and TIMP-inhibited MMPs are activated by SIBLINGs, which bind exclusively to MMPs [50]. OPN/MMP-3, DMP-1/MMP-9, and BSP/MMP-2 are examples of the known partners. DSPP can be broken down by MMP-9 into dentin sialoprotein (DSP) and dentin phosphoprotein (DPP), and MMP-2 can also cleave DMP-1 to liberate physiologically active peptides [51]. There is currently no recognized MMP companion for DSPP or MEPE [52]. SIBLINGs make up the majority of the extracellular matrix proteins in dentin that have been phosphorylated, and they have been linked to mineralization and odontoblast development [53]. These two protein families' connections might give a chance for dentin healing.

## Conclusions

MMPs are a family of proteinases that are in charge of dentin repair by modulating non-collagenous proteins and signaling molecules as well as matrix formation and remodeling. These proteinases have as many different activities as the protein family itself, and they play a developmental role in both illness and healing. The available literature demonstrates that not all MMPs have the same characteristics. Some may function more destructively than others, while vice versa.

Dentin regeneration has so far been tackled in one of two ways: either by encouraging stem cells to deposit dentin or by creating scaffolds that will help mineralized tissue deposit where the dentin deficiency is. These multifarious proteins are excellent candidates for stimulating dentin regeneration because of the spatiotemporal modulation of MMP production, their multifunctionality, and their capacity to autoregulate. Future studies should concentrate on utilizing these enzymes' characteristics to encourage dentin regeneration.
